# Pharmacological validation of TDO as a target for Parkinson’s disease

**DOI:** 10.1111/febs.15721

**Published:** 2021-02-18

**Authors:** Paula Perez‐Pardo, Yvonne Grobben, Nicole Willemsen‐Seegers, Mitch Hartog, Michaela Tutone, Michelle Muller, Youri Adolfs, Ronald Jeroen Pasterkamp, Diep Vu‐Pham, Antoon M. van Doornmalen, Freek van Cauter, Joeri de Wit, Jan Gerard Sterrenburg, Joost C.M. Uitdehaag, Jos de Man, Rogier C. Buijsman, Guido J.R. Zaman, Aletta D. Kraneveld

**Affiliations:** ^1^ Division of Pharmacology Faculty of Science Utrecht Institute for Pharmaceutical Sciences Utrecht University Utrecht The Netherlands; ^2^ Netherlands Translational Research Center B.V Oss The Netherlands; ^3^ Department of Translational Neuroscience UMC Utrecht Brain Center University Medical Center Utrecht Utrecht University Utrecht The Netherlands

**Keywords:** blood–brain barrier, enzyme inhibitors, L‐tryptophan, rotenone, tryptophan 2,3‐dioxygenase

## Abstract

Parkinson’s disease patients suffer from both motor and nonmotor impairments. There is currently no cure for Parkinson’s disease, and the most commonly used treatment, levodopa, only functions as a temporary relief of motor symptoms. Inhibition of the expression of the L‐tryptophan‐catabolizing enzyme tryptophan 2,3‐dioxygenase (TDO) has been shown to inhibit aging‐related α‐synuclein toxicity in *Caenorhabditis elegans*. To evaluate TDO inhibition as a potential therapeutic strategy for Parkinson’s disease, a brain‐penetrable, small molecule TDO inhibitor was developed, referred to as NTRC 3531‐0. This compound potently inhibits human and mouse TDO in biochemical and cell‐based assays and is selective over IDO1, an evolutionary unrelated enzyme that catalyzes the same reaction. In mice, NTRC 3531‐0 increased plasma and brain L‐tryptophan levels after oral administration, demonstrating inhibition of TDO activity *in vivo*. The effect on Parkinson’s disease symptoms was evaluated in a rotenone‐induced Parkinson’s disease mouse model. A structurally dissimilar TDO inhibitor, LM10, was evaluated in parallel. Both inhibitors had beneficial effects on rotenone‐induced motor and cognitive dysfunction as well as rotenone‐induced dopaminergic cell loss and neuroinflammation in the *substantia nigra*. Moreover, both inhibitors improved intestinal transit and enhanced colon length, which indicates a reduction of the rotenone‐induced intestinal dysfunction. Consistent with this, mice treated with TDO inhibitor showed decreased expression of rotenone‐induced glial fibrillary acidic protein, which is a marker of enteric glial cells, and decreased α‐synuclein accumulation in the *enteric plexus*. Our data support TDO inhibition as a potential therapeutic strategy to decrease motor, cognitive, and gastrointestinal symptoms in Parkinson’s disease.

## Graphical abstract

Using two selective small molecule inhibitors, the inhibition of tryptophan 2,3‐dioxygenase (TDO) was validated as a potential new therapeutic approach for Parkinson's disease. In a rotenone‐induced mouse model of Parkinson's disease, TDO inhibitor treatment induced positive effects on central nervous system function. Moreover, in contrast to standard therapy, the TDO inhibitors acted peripherally on the intestinal phenotype by reducing the rotenone‐induced intestinal inflammatory response.

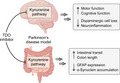

**Abbreviations**
3‐HK: 3‐hydroxykynurenine
ACMSD: aminocarboxymuconate semialdehyde decarboxylase
AUC: area under the curve
BCRP: breast cancer resistance protein
BCS: bovine calf serum
CYP: cytochrome P450
DMSO: dimethylsulfoxide
FBS: fetal bovine serum
GFAP: glial fibrillary acidic protein
IC_50_
: half‐maximal inhibitory concentration
IDO1: indoleamine 2,3‐dioxygenase
KMO: kynurenine‐3‐monooxygenase
Kp: brain‐to‐blood partition coefficient
Kp_uu_
: unbound brain‐to‐blood partition coefficient
Kyn: L‐kynurenine
LC‐MS/MS: liquid chromatography–tandem mass spectrometry
NAD: nicotine adenine dinucleotide
NFK: *N*‐formylkynurenine
NMDA: *N*‐methyl‐D‐aspartate
PK: pharmacokinetic
PD: Parkinson’s disease
pDMAB: 4‐dimethylaminobenzaldehyde
P/S: penicillin/streptomycin
QA: quinolinic acid
qPCR: quantitative real‐time PCR
SN: *substantia nigra*
SSRI: selective serotonin reuptake inhibitor
TDO: tryptophan 2,3‐dioxygenase
TH: tyrosine hydroxylase
Trp: L‐tryptophan

## Introduction

Parkinson’s disease (PD) is a chronic, progressive neurodegenerative disease characterized by motor symptoms such as bradykinesia, resting tremor, rigidity, and late postural instability. PD involves the degeneration of dopamine‐generating neurons in the *substantia nigra* (SN) [1]. Misfolding and aggregation of α‐synuclein in the remaining dopaminergic neurons results in inclusions termed Lewy bodies [2]. This pathology is also found in the peripheral autonomic nervous system, where it affects the function of various organs, including the heart and the gut [2, 3]. Moreover, several studies have described a role for neuroinflammation in PD, the hallmark of which is microgliosis [4]. In addition to the motor symptoms, PD patients often experience nonmotor symptoms, including depression [5], cognitive decline [6], and most commonly, gastrointestinal disturbances [7]. Gastrointestinal symptoms may precede the classical motor symptoms of PD by many years [8], and their occurrence in otherwise healthy people is associated with an increased risk of PD [8, 9].

To date, PD remains incurable. The most commonly used treatment is levodopa, which induces an increase in the synthesis of dopamine in the termini of the remaining dopaminergic neurons in the brain and improves motor function. However, levodopa does not prevent neurodegeneration and therefore acts only as a temporary remedy for motor symptoms [10]. Levodopa has no effect on the nonmotor symptoms of PD, which are major determinants for the quality of life of PD patients [11]. Furthermore, the long‐term use of levodopa causes inter‐dose dyskinesias [12]. Therefore, there is a need for new therapeutic strategies that alter the disease course or improve the treatment of both motor and nonmotor symptoms of PD.

Van der Goot and coworkers [13] identified the L‐tryptophan (Trp)‐catabolizing enzyme tryptophan 2,3‐dioxygenase (TDO) (EC 1.13.11.11) as a potential drug target for PD in a genome‐wide RNA interference screen for regulators of aging‐related α‐synuclein toxicity in *Caenorhabditis elegans*. Genetic and pharmacological experiments in *Drosophila melanogaster* models of Parkinson’s, Huntington’s, and Alzheimer’s disease confirmed that inhibition of TDO can ameliorate neurodegeneration [14, 15].

**Fig. 1:**
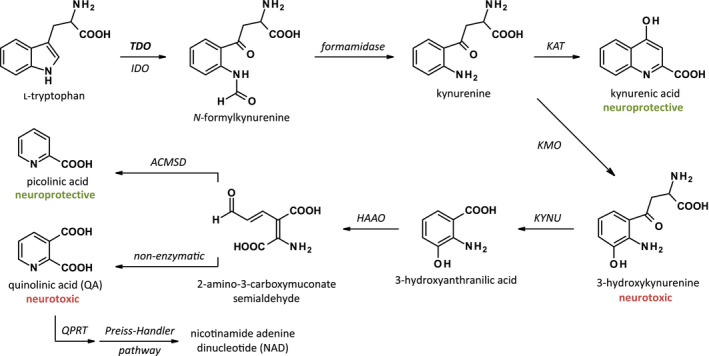
Diagram of the enzymes and metabolites of the kynurenine pathway. ACMSD, aminocarboxymuconate semialdehyde decarboxylase; HAAO, 3‐hydroxyanthranilic acid oxygenase; IDO, indoleamine 2,3‐dioxygenase; KAT, kynurenine aminotransferase; KMO, kynurenine 3‐monooxygenase; KYNU, kynureninase; NAD, nicotinamide adenine dinucleotide; QPRT, quinolinate phosphoribosyltransferase; TDO, tryptophan 2,3‐dioxygenase.

TDO catalyzes the first, rate‐limiting step of the catabolism of the amino acid Trp in the kynurenine pathway, an enzymatic cascade responsible for the synthesis of nicotine adenine dinucleotide (NAD) and NAD phosphate (Fig. 1). [16]. In the human brain, 95% of Trp is metabolized via the kynurenine pathway, while the remaining Trp is used for the synthesis of serotonin or proteins. The kynurenine pathway of Trp metabolism produces neuroprotective as well as neurotoxic metabolites (Fig. 1). PD, like several other neurodegenerative diseases, has been associated with an imbalance between these neuroactive metabolites [17, 18, 19, 20, 21]. There is no genetic evidence for a direct role of TDO or other enzymes of the pathway in the development of PD. However, a locus in close proximity to the gene encoding aminocarboxymuconate semialdehyde decarboxylase (ACMSD), a downstream enzyme in the kynurenine pathway (Fig. 1), is associated with an increased risk of sporadic PD, suggesting that ACMSD may influence the pathogenesis of PD [22, 23].

Modulation of the kynurenine pathway with small molecule inhibitors has been explored already for more than two decades for neurodegenerative diseases [21]. There have been several drug discovery programs on inhibitors of kynurenine‐3‐monooxygenase (KMO), the enzyme responsible for the generation of the neurotoxic metabolite 3‐hydroxykynurenine (3‐HK) [24]. While none of these efforts have yet resulted in compounds investigated in the clinic due to lack of brain penetration [25], a non‐blood–brain barrier‐penetrating KMO inhibitor was shown to significantly improve Alzheimer’s disease symptoms and slow the disease progression [24].

To evaluate TDO inhibition as a potential therapeutic strategy for PD, a brain‐penetrable, small molecule inhibitor of TDO was developed (NTRC 3531‐0). To investigate the effects on symptoms associated with PD, the compound was evaluated in a rotenone‐induced mouse model of PD [26, 27, 28]. The natural product rotenone is a pesticide that interferes with mitochondrial function [26]. Rotenone exposure is associated with an increased risk of PD in humans [26], and many of the hallmarks of the disease in humans have been reproduced in the rotenone model. These include the loss of dopaminergic cell bodies in the SN and α‐synuclein aggregation, but also gastrointestinal dysfunction, which is thought to arise from a bi‐directional communication system between the central nervous system and the gastrointestinal tract, referred to as the gut‐brain axis [26, 27, 28]. In parallel with the evaluation of NTRC 3531‐0, a chemically different TDO inhibitor, LM10, published by the Ludwig Cancer Center [29, 30], was tested in the same animal model. LM10 was previously shown to effectively inhibit the growth of TDO‐expressing tumors [30]. We observed that both TDO inhibitors inhibited the development of motor, cognitive, and intestinal dysfunctions. Moreover, TDO inhibitor treatment was associated with a decreased loss of dopaminergic cells and decreased microglia activation in the SN, as well as with reduced glial cell marker expression and reduced accumulation of α‐synuclein in the enteric nerves of the intestinal tract.

## Results

### *In vitro* characterization of a novel, selective TDO inhibitor

TDO catalyzes the oxidation of Trp, resulting in the formation of *N*‐formylkynurenine (NFK) [16]. To identify small molecule inhibitors of TDO, a library of 87,000 synthetic low‐molecular‐weight molecules was screened with a biochemical assay, making use of recombinant human TDO (hTDO) and a fluorescent probe that specifically binds to NFK [31]. Hit compounds were tested for selectivity against human indoleamine 2,3‐dioxygenase (hIDO1) (EC 1.13.11.42), an evolutionary unrelated enzyme that catalyzes the same reaction, using a similar biochemical assay [31]. Cellular activity was determined in cell lines derived from the human embryonic kidney cell line HEK‐293, stably overexpressing the full‐length human *TDO2* or *IDO1* cDNA.

**Fig. 2:**
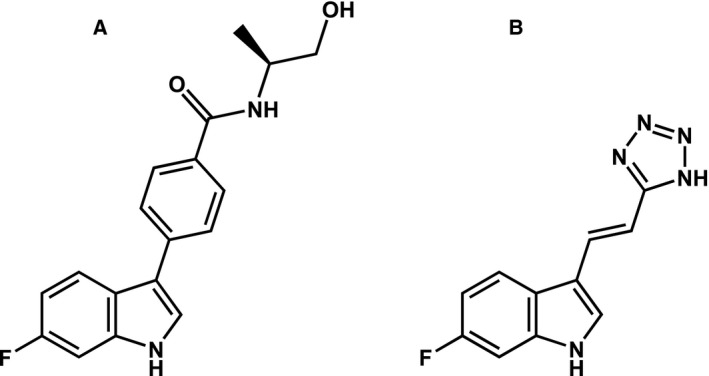
Chemical structure of NTRC 3531‐0 (A) and LM10 (B).

Optimization of one of the screening hits resulted in NTRC 3531‐0, a potent and selective TDO inhibitor with a 3‐phenyl‐1*H*‐indole scaffold (Fig. 2) [32]. NTRC 3531‐0 inhibited hTDO with a half‐maximal inhibitory concentration (IC_50_) of 490 nM in the biochemical assay, and with an IC_50_ of 816 nM in *hTDO2*‐overexpressing HEK‐293 cells (HEK‐hTDO) (Table 1). The compound also inhibited the conversion of Trp to NFK in the colon carcinoma cell line SW48 (Table 1), which constitutively expresses TDO [31].

**Table 1:** Potency (IC_50_) in nm of TDO inhibitors NTRC 3531‐0 and LM10 in biochemical and cell‐based assays for human (h) and mouse (m) TDO or IDO1. Confidence intervals and number of experimental replicates (n) are given within brackets

Assay	NTRC 3531‐0	LM10
hTDO	490 (461–520) (*n* = 76)	716 (491–1040) (*n* = 8)
hIDO1	<20% inhibition @ 31 600 (*n* = 72)	<20% inhibition @ 31 600 (n = 6)
HEK‐hTDO	816 (*n* = 2)	33% inhibition @ 31 600 (*n* = 2)
HEK‐hIDO1	20 700 (*n* = 2)	<20% inhibition @ 31 600 (*n* = 2)
SW48 (hTDO)	220 (184–262) (*n* = 13)	31 600 (22 800–43 800) (*n* = 5)^a^
mTDO	846 (728–983) (*n* = 3)	1070 (732–1570) (*n* = 3)
GL‐261‐mTDO	214 (*n* = 2)	42% inhibition @ 31 600 (*n* = 2)

^a^ Values are based on extrapolation above 31,600 nm for three out of five experiments.

NTRC 3531‐0 was inactive on hIDO1 in the biochemical assay at the highest tested concentration (*i.e.*, 31.6 µM), and it inhibited Trp metabolism in human *IDO1*‐overexpressing HEK‐293 cells (HEK‐hIDO1) with an IC_50_ of 20.7 µM (Table 1). In biochemical assays, NTRC 3531‐0 is therefore more than 60 times selective for hTDO over hIDO1, and in the HEK‐293 cell‐based assays at least 25 times. NTRC 3531‐0 also inhibited mouse TDO (mTDO) in a biochemical assay and in a cellular assay based on a mouse glioma GL‐261 cell line stably overexpressing the full‐length mouse *TDO2* cDNA (GL‐261‐mTDO) (Table 1). NTRC 3531‐0 was slightly less potent on mTDO compared to hTDO in the biochemical assays (Table 1). Inhibitory potency in the GL‐261‐mTDO cellular assay was similar to that in the SW48 cells and was four times higher compared to the potency in HEK‐hTDO cells (Table 1).

Since TDO contains a heme center, similar to cytochrome P450 (CYP) enzymes, we evaluated the selectivity of NTRC 3531‐0 on a panel of seven CYP enzymes, that is, CYP1A2, CYP2B6, CYP2C8, CYP2C9, CYP2C19, CYP2D6, and CYP3A4. NTRC 3531‐0 inhibited CYP2C8 with an IC_50_ of 16.3 µM, and was inactive against all other CYPs (IC_50_ > 31.6 µM). These data indicate that NTRC 3531‐0 selectively interacts with the active site of TDO.

In our biochemical assays, NTRC 3531‐0 was slightly more potent on hTDO than LM10 (Table 1) [29]. However, LM10 showed a considerably lower potency in our cellular assays, with respectively 33% and 50% inhibition at a concentration of 31.6 μM in the HEK‐hTDO and SW48 cells (Table 1). In both the biochemical and cell‐based assays, the activity of LM10 on mTDO was similar to that on hTDO (Table 1).

### Pharmacokinetics and brain penetration

To determine whether NTRC 3531‐0 could potentially enter the brain, a bi‐directional transport assay was performed with Madin‐Darby canine kidney (MDCK) cells overexpressing the human *ABCB1* gene‐encoded MDR1 P‐glycoprotein (MDCK‐MDR1) at Cyprotex (Macclesfield, UK) [33]. MDCK‐MDR1 cells form a confluent monolayer in Transwell^®^ cell culture plates (Corning) and express active MDR1 P‐glycoprotein at the apical side. The cell model is a surrogate model of the brain capillary endothelial cell layer that constitutes the blood–brain barrier [34]. The transport of NTRC 3531‐0 from the apical (A) to the basolateral (B) side of the monolayer (*P*_app_, _A to B_), and from basolateral (B) to apical (A) (*P*_app_, _B to A_), was determined by measuring compound levels in the two compartments of the Transwell plates by liquid chromatography–tandem mass spectrometry (LC‐MS/MS) (Table 2). Transport assays were performed in the absence and presence of the P‐glycoprotein inhibitor elacridar.

**Table 2:** Bi‐directional transport through a monolayer of human MDR1 P‐glycoprotein‐overexpressing MDCK cells

	Elacridar	*P*_app, A to B_ (10^‐6^ cm∙s^−1^)	*P*_app, B to A_ (10^‐6^ cm∙s^−1^)	Efflux ratio
NTRC 3531‐0	−	15.7	52.0	3.32
NTRC 3531‐0	+	26.0	14.0	0.54
LM10	−	5.63	1.58	0.28
LM10	+	3.50	1.27	0.36

NTRC 3531‐0 showed passage through the MDCK‐MDR1 cell layer from A to B, indicating that it may cross the blood–brain barrier. However, efflux from B to A was higher (P_app_, _B to A_ > P_app_, _A to B_), resulting in an efflux ratio of 3.32 (Table 2), indicating that NTRC 3531‐0 could be a weak substrate of P‐glycoprotein. In agreement with this, B to A transport and the efflux ratio strongly decreased in the presence of elacridar. In contrast, LM10 showed higher transport from A to B than from B to A (Table 2), which is indicative of an active uptake mechanism present in the MDCK cells. Elacridar had no effect on transport or efflux, indicating that LM10 is not a P‐glycoprotein substrate (Table 2).

**Fig. 3:**
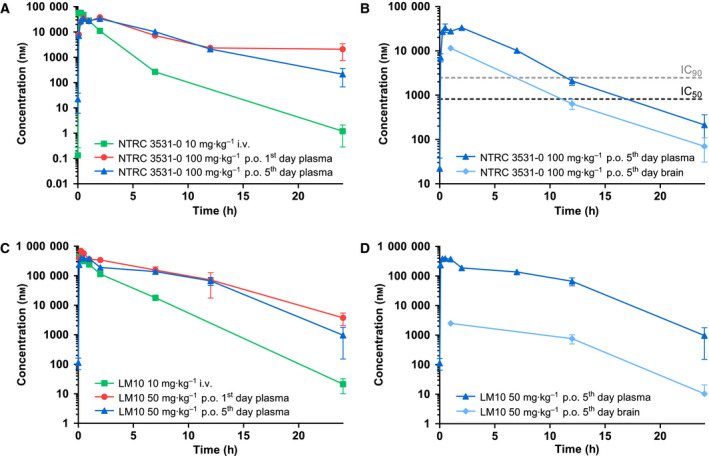
Pharmacokinetics of NTRC 3531‐0 and LM10 in plasma and brain. (A) Plasma levels in time of NTRC 3531‐0 measured after single i.v. administration of 10 mg·kg^−1^, single p.o. administration of 100 mg·kg^−1^, or after the 5^th^ dose of a 5‐day consecutive, once daily p.o. dosing of 100 mg·kg^−1^. (B) Levels of NTRC 3531‐0 in plasma and brain after the 5^th^ dose. The horizontal dashed lines correspond to the IC_50_ and IC_90_ concentrations of NTRC 3531‐0 in the HEK‐hTDO assay. (C) Plasma levels in time of LM10 measured after single i.v. administration of 10 mg·kg^−1^, single p.o. administration of 50 mg·kg^−1^, or after the 5^th^ dose of a of 5‐day consecutive, once daily p.o. dosing of 50 mg·kg^−1^. (D) Levels of LM10 in plasma and brain after the 5^th^ dose. Plasma and brain levels are expressed as mean ± SEM of 3 mice per time point and dosing group.

*In vivo* pharmacokinetic (PK) properties and brain penetration of NTRC 3531‐0 were determined in male C57BL/6NCrl mice (Fig. 3A,B). Oral bioavailability (F) was determined by comparing plasma levels after a single per oral (p.o.) administration of 100 mg·kg^−1^ inhibitor with the levels after a single intravenous (i.v.) dose of 10 mg·kg^−1^. Plasma and brain levels at steady state were determined after administration of 100 mg·kg^−1^ inhibitor for five consecutive days. Compound concentrations in plasma samples and brain homogenates were measured by LC‐MS/MS. Permeability across the blood–brain barrier was determined by calculation of the brain‐to‐blood partition coefficient (Kp) [35], based on the areas under the curves (AUCs) determined after 5 days dosing. NTRC 3531‐0 had an oral bioavailability of 22% and a high *C*_max_ (Table 3). The Kp was 0.37 after repeated dosing. The unbound brain‐to‐plasma partition coefficient (Kp_uu_) [35] of NTRC 3531‐0 was 0.067 (Table 3).

**Table 3:** *In vivo* pharmacokinetic profile of the TDO inhibitors NTRC 3531‐0 and LM10 in mice

	NTRC 3531‐0			LM10		
Administration route	i.v.	single p.o.	5‐day p.o.	i.v.	single p.o.	5‐day p.o.
Dose (mg∙kg^−1^)	10	100	100	10	50	50
*C*_max_ (μmol∙L^−1^)	56.5	37.5	33.2	427	688	391
*t*_½_ (h)	0.903	2.49	3.13	1.77	3.09	2.35
AUC_plasma_ (h∙μmol∙L^−1^)	93.2	207	210	985	3100	2360
AUC_brain_ (h∙μmol∙L^−1^)		84.5	77.0		18.5	23.8
Kp		0.41	0.37		0.006	0.010
Kp_uu_		0.074	0.067		0.012	0.020
*F* (%)		22.3			63.1	

AUC, area under the curve; C_max_, maximum plasma concentration; *F*, oral bioavailability; Kp, brain‐to‐plasma partition coefficient; Kp_uu_, unbound brain‐to‐plasma partition coefficient; *t*_½_, half‐life.

An additional PK study was performed in C57BL/6NCrl mice for LM10, which was dosed at 50 mg·kg^−1^ p.o. and 10 mg·kg^−1^ i.v. (Fig. 3C,D). LM10 had a good oral bioavailability of 63% and reached more than ten times higher plasma levels compared to NTRC 3531‐0, at two times lower dose (Table 3). However, the Kp of LM10 was 0.010 after repeated dosing. The Kp_uu_ of LM10 was 0.020, indicating a considerably less efficient brain penetration than NTRC 3531‐0.

**Fig. 4:**
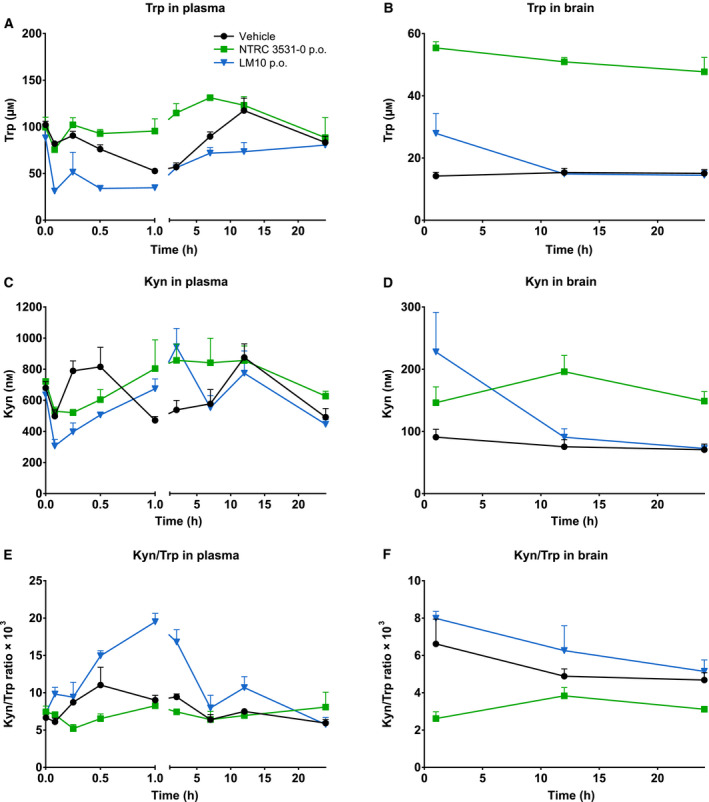
*In vivo* effect of NTRC 3531‐0 on L‐tryptophan (Trp) and L‐kynurenine (Kyn) levels. (A,B) Plasma and brain levels in time of Trp after single p.o. administration of 100 mg·kg^−1^ NTRC 3531‐0 or 50 mg·kg^−1^ LM10. Two‐way ANOVA showed an overall effect of TDO inhibitor treatment on Trp levels in both plasma (*P* < 0.0001) and brain (*P* < 0.0001), an overall effect of time on Trp levels in plasma (*P* < 0.0001) and brain (*P* < 0.001), and an interaction effect between TDO inhibitor treatment and time in plasma (*P* < 0.0001). (C,D) Plasma and brain levels of Kyn. Two‐way ANOVA showed an overall effect of TDO inhibitor treatment on Kyn levels in brain (*P* < 0.01), an overall effect of time on Kyn levels in plasma (*P* < 0.0001) and brain (*P* < 0.01), and an interaction effect between TDO inhibitor treatment and time in plasma (*P* < 0.0001) and brain (*P* < 0.01). Basal levels of Trp and Kyn were determined after treatment with vehicle. (E,F) Kyn/Trp ratio in plasma and brain. Levels after single treatment were determined in naive mice, which regained access to feed 2 h after dosing. Plasma and brain levels are expressed as mean ± SEM of 3 mice per time point and dosing group.

To determine whether the inhibitors modulated TDO activity *in vivo*, the concentrations of Trp and L‐kynurenine (Kyn) were measured in the plasma and brain samples of the PK studies using LC‐MS/MS (Fig. 4). Oral administration of NTRC 3531‐0 resulted in an increase in the concentration of Trp in plasma (1 h, *P* < 0.01; 2 h, *P* < 0.0001; 7 h, *P* < 0.01) as well as in brain (*P* < 0.0001 at 1, 12 and 24 h) compared to mice treated with vehicle (Fig. 4A,B), which conforms with the inhibition of TDO activity. Kyn levels were found to be slightly decreased in plasma at early timepoints (*i.e.*, 0.25 and 0.5 h), although no statistical significance was reached, while the levels subsequently increased in both plasma (1 and 2 h, *P* < 0.05) and brain (12 h, *P* < 0.01; 24 h, *P* < 0.05) (Fig. 4C,D). In contrast, no increase in Trp levels was detected after administration of LM10 (Fig. 4A,B), while the plasma Kyn levels showed a similar pattern as observed for NTRC 3531‐0 treatment, but only the initial decrease in Kyn levels was found to be significant (0.25 h, *P* < 0.01; 0.5 h, *P* < 0.05) (Fig. 4C).

## Effects on motor and cognitive functions in rotenone model

**Fig. 5:**
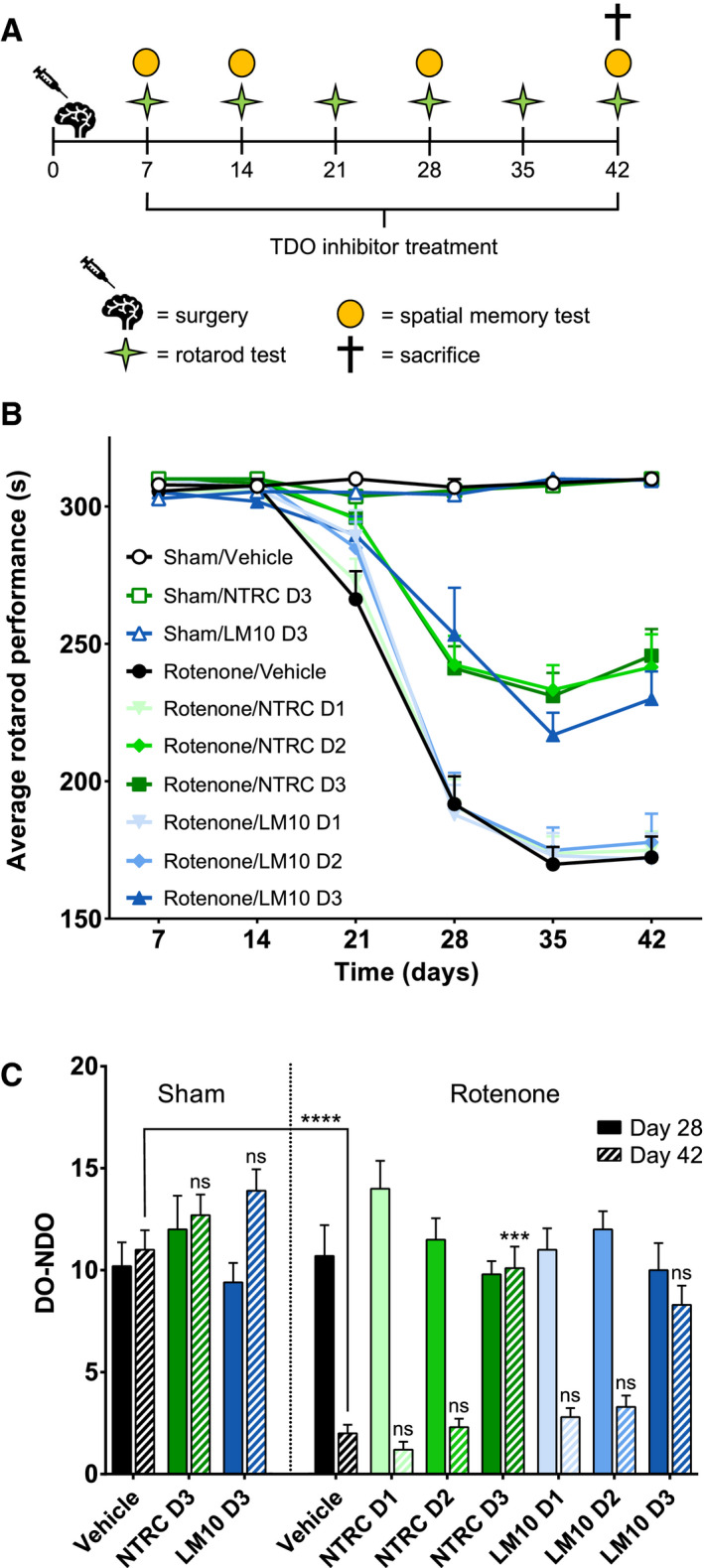
Evaluation of motor and cognitive function of mice treated with rotenone and TDO inhibitors. (A) Experimental set‐up of the study with the numbers representing the days after surgery. PD was induced in mice by infusing rotenone in the striatum of the mice. Starting at day 7 after the operation, mice were treated once daily with an oral gavage of vehicle, NTRC 3531‐0 (D1: 25 mg·kg^−1^; D2: 50 mg·kg^−1^; D3: 100 mg·kg^−1^) or LM10 (D1: 12.5 mg·kg^−1^; D2: 25 mg·kg^−1^; D3: 50 mg·kg^−1^). (B) Rotarod performance of mice treated with rotenone and TDO inhibitors. Two‐way ANOVA showed an overall effect of rotenone injection on rotarod performance starting from day 21 (*P* < 0.0001). Repeated measures demonstrated that rotenone‐treated mice developed motor problems with time when compared to sham‐operated mice (interaction effect rotenone and time) (*P* < 0.0001). (C) Effect of TDO inhibitors on spatial memory at day 28 and day 42. Spatial discrimination is determined by comparing the time mice spent to explore a nondisplaced object (NDO) or a displaced object (DO), upon returning into a cage. Two‐way ANOVA showed an overall effect of rotenone injection on day 42 (*P* < 0.0001). *N* = 10 mice per group for panels B and C. Results are expressed as mean ± SEM. Indications of significance above individual bars represent the comparison to the respective vehicle control of the sham‐operated or rotenone‐injected groups. ns, not significant (*P* > 0.05); ***P < 0.001; ****P < 0.0001.

To evaluate TDO inhibition as a potential therapeutic strategy for PD, NTRC 3531‐0 and LM10 were evaluated in parallel in a rotenone‐induced mouse model of PD [26, 27, 28]. To investigate the effects of TDO inhibition, both compounds were orally administered once daily from day 7 after induction of the PD model (Fig. 5A). To determine the effect of TDO inhibitor treatment on rotenone‐induced motor dysfunction, the rotarod test was performed (Fig. 5A,B). In this test, the time spent on an accelerating wheel was used to analyze motor function. Rotenone‐infused, vehicle‐treated mice demonstrated a significant motor dysfunction starting 21 days after surgery when compared to sham‐operated, vehicle‐treated animals (*P* < 0.01) (Fig. 5B). The highest dose of both TDO inhibitors did not affect motor function in sham‐operated mice (Fig. 5B). However, both TDO inhibitors improved the motor function of rotenone‐treated mice (Fig. 5B). Specifically, treatment with NTRC 3531‐0 at the two highest doses (50 mg·kg^−1^, *P* < 0.01 at day 35; 100 mg·kg^−1^, *P* < 0.01), and LM10 at the highest dose (50 mg·kg^−1^, *P* < 0.05) resulted in a significantly improved motor function (Fig. 5B).

The cognitive function of the mice was evaluated in a spatial memory test [36]. In this test, the mice were placed in a cage where they were allowed to habituate to the configuration of a number of objects. Subsequently, the object exploration behavior of the mice was evaluated after relocation of two of the objects in the cage. Sham‐operated, vehicle‐treated mice were able to selectively react to a spatial change during the entire course of the animal experiment, that is, they selectively re‐explored the displaced objects compared to the nondisplaced objects (Fig. 5C). Both TDO inhibitors did not affect spatial memory in sham‐operated mice (Fig. 5C). The spatial recognition of animals infused with rotenone was significantly affected at 42 days after surgery when compared to sham‐operated, vehicle‐treated animals (*P* < 0.0001). At the same time point, however, there was significantly less rotenone‐induced spatial recognition loss in mice treated with the highest dose of NTRC 3531‐0 (*P* < 0.001). Results for mice treated with the highest dose of LM10 showed a similar trend, but were not statistically significant (Fig. 5C).

**Fig. 6:**
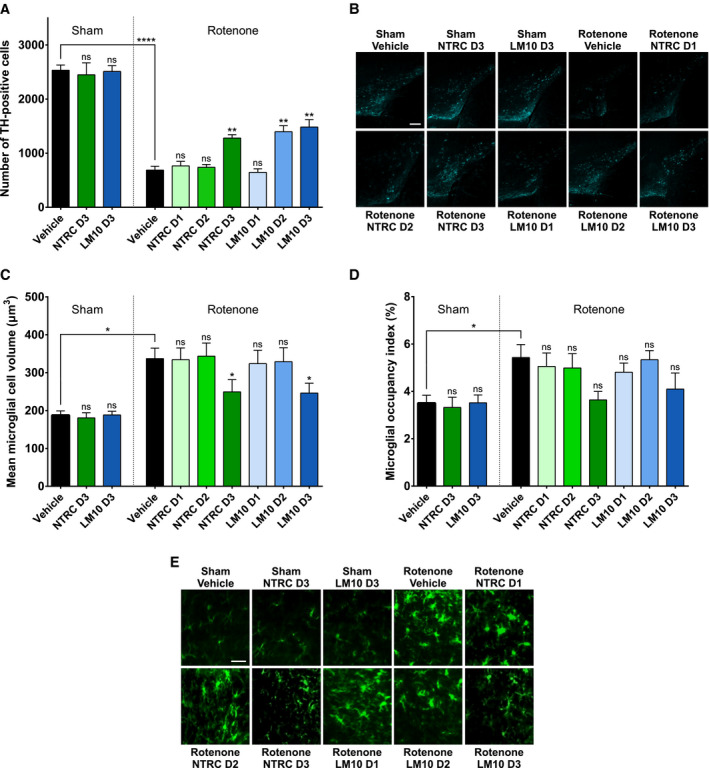
Evaluation of dopaminergic cell loss and neuroinflammation in mice treated with rotenone and TDO inhibitors. (A) Effect of rotenone and TDO inhibitors on the number of dopaminergic cells (tyrosine hydroxylase‐positive cells) in the *substantia nigra*. Two‐way ANOVA showed an overall effect of rotenone injection on the number of dopaminergic neurons in the *substantia nigra* (*P* < 0.0001). (B) Representative 2D images of anti‐TH‐labeled dopaminergic cells in the cleared brains of mice treated with rotenone and TDO inhibitors (scale bar: 250 μm). (C) Effect of rotenone and TDO inhibitors on the volume of microglia in the *substantia nigra*. Two‐way ANOVA showed an overall effect of rotenone injection on the volume of the microglia (*P* < 0.0001). (D) Effect of rotenone and TDO inhibitors on the space occupied by microglia in the *substantia nigra*. Rotenone injection decreased the space occupied by microglia compared to sham‐operated mice. The labeling of the groups is the same as listed for Fig. 5A. *N* = 4 mice per group for all groups in panels A, C and D, except for the sham/NTRC D3 and rotenone/LM10 D3 groups with *N* = 3, and the sham/LM10 D3 group with *N* = 5. Results are expressed as mean ± SEM. Indications of significance above individual bars represent the comparison to the respective vehicle control of the sham‐operated or rotenone‐injected groups. ns, not significant (*P* > 0.05); *P < 0.05; **P < 0.01; ****P < 0.0001. (E) Representative 2D images of anti‐Iba1‐labeled microglial cells in the cleared brains of mice treated with rotenone and TDO inhibitors (scale bar: 30 μm).

To determine the effect of TDO inhibitor treatment on rotenone‐induced dopaminergic cell loss, the number of dopaminergic cells in the SN of mice was quantified by immunolabeling of tyrosine hydroxylase (TH)‐positive cells and 3D image analysis [37]. Infusion with rotenone resulted in a reduction of the number of TH‐positive cell in the SN (*P* < 0.0001) when compared to sham‐operated, vehicle‐treated mice (Fig. 6A,B). Both TDO inhibitors did not affect dopaminergic cell counts in the SN of sham‐operated mice. NTRC 3531‐0, at the highest dose, and LM10, at the two highest doses, decreased the rotenone‐induced loss of TH‐positive cells in the SN (*P* < 0.01 for both inhibitors) (Fig. 6A,B). This demonstrates that TDO inhibitor treatment decreases neurodegeneration.

To evaluate the effect of TDO inhibitor treatment on rotenone‐induced neuroinflammation, the volume of microglial cells and the space occupied by these cells in the SN was measured by immunolabeling of Iba1 followed by 3D image analysis. Microglia are morphologically and functionally dynamic cells, and their activation is associated with an increased cell body size and decreased number of branches, reflecting the morphological transition from a ramified ‘resting’ to an activated phagocytic ameboid‐like phenotype [38]. Infusion with rotenone resulted in an increased mean volume of the microglia in the SN, which is indicative for microglia cell body enlargement, when compared to sham‐operated, vehicle‐treated mice (*P* < 0.05) (Fig. 6C,E). Both TDO inhibitors did not affect the mean microglia volume in sham‐operated mice. However, at the highest dose, both NTRC 3531‐0 and LM10 reduced the mean microglia volume in rotenone‐treated mice (*P* < 0.05 for both inhibitors) (Fig. 6C). This indicates that TDO inhibitor treatment decreases the rotenone‐induced neuroinflammation in the SN. Infusion of mice with rotenone additionally resulted in an increase of the total space occupied by microglia in the SN when compared to sham‐operated, vehicle‐treated mice (*P* < 0.05) (Fig. 6D,E). Treatment with the TDO inhibitors resulted in a similar trend as observed for the microglia volume, although the effects were not statistically significant (Fig. 6D).

## Effects on intestinal phenotype in rotenone model

**Fig. 7:**
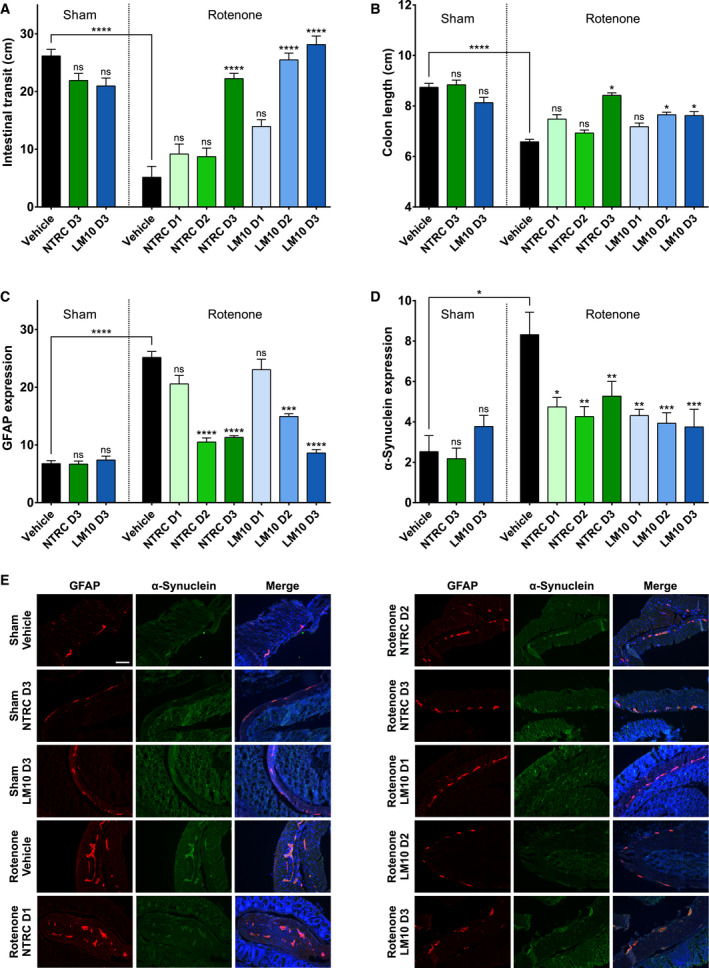
Gastrointestinal phenotype of mice treated with rotenone and TDO inhibitor. (A) Transit time. Two‐way ANOVA showed an overall effect of rotenone injection on intestinal transit (*P* < 0.0001) and an interaction effect between rotenone and TDO inhibitor treatment (*P* < 0.05). (B) Colon length. Two‐way ANOVA showed an overall effect of rotenone injection on colon length (*P* < 0.0001). (C) Expression of glial fibrillary acidic protein (GFAP) in enteric glial cells. Two‐way ANOVA showed an overall effect of rotenone injection on GFAP expression (*P* < 0.001). (D) α‐Synuclein expression in colonic tissue. The labeling of the groups is the same as listed for Fig. 5A. *N* = 10 mice per group for all groups in panels A to D, except for the sham/LM10 D3 and rotenone/vehicle groups in panel A with *N* = 9, the rotenone/LM10 D1 and D2 groups in panel A with *N* = 8, and the rotenone/NTRC D3 group in panel C with *N* = 9. Results are expressed as mean ± SEM. Indications of significance above individual bars represent the comparison to the respective vehicle control of the sham‐operated or rotenone‐injected groups. ns, not significant (*P* > 0.05); *P < 0.05; **P < 0.01; ***P < 0.001; ****P < 0.0001. (E) Representative images of GFAP expression (red) as a marker for enteric glial cells, α‐synuclein expression (green) and DAPI staining (blue) in the colonic tissue of mice treated with rotenone and TDO inhibitors (scale bar: 100 μm).

To evaluate the effect of TDO inhibitor treatment on rotenone‐induced gastrointestinal dysfunction, the intestinal transit of a dye (Evans blue) was determined. The dye was injected intragastrically, thirty minutes before the sacrifice of the mice. Intestinal transit was measured as the distance travelled by the dye from the pylorus to the most distal part of the colon. Infusion with rotenone resulted in a significant delay of intestinal transit when compared to sham‐operated, vehicle‐treated mice (*P* < 0.0001) (Fig. 7A). Both TDO inhibitors at the highest dose did not affect the intestinal transit time of sham‐operated mice. Treatment with the highest dose of NTRC 3531‐0, and the two highest doses of LM10 improved the intestinal transit of the PD mice (*P* < 0.0001 for all) (Fig. 7A). This suggests that treatment with TDO inhibitor restores the PD‐associated disturbed gastrointestinal function.

The effect of rotenone and inhibitor treatment on intestinal inflammation was determined by measuring the colon length of the mice. Infusion with rotenone resulted in a decreased colon length (*P* < 0.0001) when compared to sham‐operated, vehicle‐treated mice, which is indicative of an inflammatory response (Fig. 7B). Treatment with the highest dose of NTRC 3531‐0, and the two highest doses of LM10 reduced the rotenone‐induced intestinal inflammatory response (*P* < 0.05 for all) (Fig. 7B).

Parkinson’s disease is associated with the activation of glial cells in the enteric nervous system [7]. Furthermore, aggregates of α‐synuclein have been observed in the enteric nervous system of PD patients [7]. The expression of glial fibrillary acidic protein (GFAP), a marker of enteric glial cells, and of α‐synuclein was determined by immunofluorescent staining of fixed sections of the colons of the sacrificed mice. Infusion with rotenone in the striatum resulted in an increased expression of GFAP (*P* < 0.0001) (Fig. 7C), and an increased accumulation of α‐synuclein in the *enteric plexus* of the colon (*P* < 0.05) when compared to sham‐operated, vehicle‐treated mice (Fig. 7D). Treatment with either NTRC 3531‐0 or LM10 at the highest two doses resulted in a decrease of GFAP staining (NTRC 3531‐0: *P* < 0.0001 for both doses; LM10: 25 mg·kg^−1^, *P* < 0.001; 50 mg·kg^−1^, *P* < 0.0001) (Fig. 7C,E), and a decrease in the expression of α‐synuclein in the *enteric plexus* of rotenone‐treated mice at all inhibitor doses (NTRC 3531‐0: 25 mg·kg^−1^, *P* < 0.05; 50 and 100 mg·kg^−1^, *P* < 0.01; LM10: 12.5 mg·kg^−1^, *P* < 0.01; 25 and 50 mg·kg^−1^, *P* < 0.001) (Fig. 7D,E). These results are consistent with the restoration of the gastrointestinal function determined by measurement of the intestinal transit and the colon length. Moreover, the results demonstrate that the TDO inhibitors do not only have positive effects on central nervous system function, but also act peripherally on the intestinal phenotype in the rotenone model.

## Discussion

PD has been associated with an imbalance of neurotoxic and neuroprotective metabolites in the kynurenine pathway [17, 18, 19, 20, 21]. Previous studies have shown that downregulation of TDO expression by RNA interference techniques in the invertebrate model species *Caenorhabditis elegans* and *Drosophila melanogaster* reduced α‐synuclein proteotoxicity [13, 15]. Formation of α‐synuclein aggregates is one of the pathological hallmarks of PD in humans [2]. Here we show for the first time in a relevant mammalian model for PD that pharmacological inhibition of TDO decreases the development of motor and nonmotor symptoms associated with the disease in humans. PD symptoms were induced by intrastriatal infusion of rotenone, and oral treatment with TDO inhibitors was started seven days after disease induction. Two chemically and pharmacologically distinct TDO inhibitors were used, NTRC 3531‐0 and LM10, which induced similar positive effects in the rotenone model. TDO inhibitor treatment was effective in reducing motor and cognitive deficits, as well as in reducing gastrointestinal dysfunction. Immunolabeling and 3D image analysis of TH‐positive cells and Iba1‐positive cells indicated that TDO inhibitor treatment decreased the rotenone‐induced loss of dopaminergic neurons and the activation of microglia in the SN. Furthermore, immunofluorescent staining of colon tissue showed that TDO inhibitor treatment reduced glial cell activation and α‐synuclein accumulation in the *enteric plexus*.

Though the restorative effects of both inhibitors were significant, they were partial. *In vivo* efficacy may have been limited by potency, or bioavailability. Assuming that at least 90% target inhibition is required for efficacy, the levels of NTRC 3531‐0 were efficacious for only half of the time between the dose administrations. Suboptimal dosing may also provide an explanation for the saturating effect seen in the rotarod test, since the 50 and 100 mg·kg^−1^ doses of NTRC 3531‐0 were equally effective. To improve the *in vivo* efficacy, derivatives of NTRC 3531‐0 are needed with significantly higher activity on TDO and improved pharmacokinetic properties. For LM10, relationships between target occupancy and efficacy could not be determined because of its low cellular potency. Nevertheless, LM10 showed similar or even stronger effects than NTRC 3531‐0 on most phenotypes in the rotenone model.

LM10 has previously been described to be effective in tumor models [30], and was originally reported as a high‐nanomolar potent inhibitor in hTDO‐overexpressing HEK‐293 cells [30]. Our cellular assays have indicated a considerably lower cellular potency of LM10, which is in agreement with a more recent publication reporting an IC_50_ of 24 μm in the constitutively TDO‐expressing human glioblastoma A172 cell line [39]. In the PK studies, we have observed that LM10, despite its high plasma levels, does not induce the expected increase in plasma Trp levels, which may be correlated with the low cellular activity of LM10. A further lack of the modulation of Trp and Kyn levels in the brain can be explained by the poor brain penetration of LM10. Nonetheless, the plasma Kyn levels showed a relatively similar pattern in time as that observed upon treatment with NTRC 3531‐0. While the initial decrease in Kyn levels would conform with the inhibition of TDO activity, the subsequent increase would seemingly contradict this. Nonetheless, this observation is in agreement with increased plasma Kyn levels observed in a TDO inhibitor‐treated tumor mouse model and in *TDO2* knock‐out mice [39, 40]. In the former study, plasma Trp and Kyn levels were studied at 2, 8, and 16 h after TDO inhibitor treatment [39]. The highest increase in Trp levels relative to vehicle‐treated mice was found at 2 h, after which the levels decreased. For Kyn, increased levels were found at both 2 and 8 h, but not at 16 h [39]. These observations are both in agreement with the observed patterns for NTRC 3531‐0. While the initial decrease in Kyn levels at the earlier timepoints may be attributed to inhibition of TDO activity, Schramme and coworkers suggested that increased Trp levels may induce IDO1 activity in peripheral IDO1‐expressing cells [39], resulting in increased Kyn levels in plasma at later timepoints. While increases in Kyn levels would expectedly nullify the effect of TDO inhibition, we believe that local changes in kynurenine pathway metabolite levels, for instance in specific cell types, or brain regions in which IDO1 is not expressed, may underlie the beneficial effects of TDO inhibitor treatment observed in the PD model, although this would require further study. Moreover, the mechanism by which LM10 induces the beneficial effect in the PD model in the absence of clear *in vivo* modulation of Kyn and Trp levels requires further understanding.

The mechanism through which TDO inhibitor treatment results in positive effects on rotenone‐induced central and peripheral phenotypes remains to be elucidated. It is important to note the differences in *in vivo* PK properties of NTRC 3531‐0 and LM10. LM10 reached very high levels in plasma after p.o. administration. Based on results obtained with an MDCK‐MDR1 transporter assay, LM10 was expected to reach high levels in brain as well. However, the concentration of LM10 measured in the brain at steady state (*i.e.*, the AUC after 5‐day p.o. dosing) was only 1% of the concentration in plasma. Although the amount of blood in the brain was reduced by exsanguination of the mice before isolation of the brains, it cannot be excluded that the low amount of LM10 measured in the brain samples originates from the blood instead of the brain tissue, since blood accounts for approximately 3 to 6% of the total brain volume in mice [41, 42]. The contribution of brain LM10 to its efficacy is therefore difficult to determine. Moreover, although the results with the MDCK‐MDR1 assay indicate that LM10 is not a substrate of P‐glycoprotein, passage across the blood–brain barrier may be impaired by another drug transporter, such as the breast cancer resistance protein (BCRP) [43]. However, BCRP and P‐glycoprotein have overlapping substrates, which implies that substrates of BCRP are often also substrates of P‐glycoprotein [44]. The presence of an acidic moiety in LM10 most likely precludes blood–brain barrier penetration due to the fact that it is mostly ionized in systemic circulation [35].

The total concentration of NTRC 3531‐0 measured in brain at steady state was 37% of the concentration in plasma (Kp of 0.37). NTRC 3531‐0 did show higher plasma protein and brain tissue binding compared to LM10, but the Kp_uu_ was still higher compared to LM10. Therefore, NTRC 3531‐0 is most suitable to be used as a pharmacological tool compound to study the functions of TDO both in the brain and in the periphery.

Since no compound or metabolite levels were measured in the efficacy study, it cannot be determined whether the disease‐modifying effects were mediated through an effect on the TDO enzyme expressed in the brain or in peripheral tissues, such as the liver or intestine [16]. Further studies are needed to determine to what extent the modulation of peripheral TDO activity can affect the central levels of Trp and its metabolites. Since Trp is actively transported over the blood–brain barrier [45], and several metabolites formed in the kynurenine pathway can cross the blood–brain barrier as well [46], modulation of central TDO activity may not be required. This could explain the beneficial effects observed for LM10 in the PD model in the absence of efficient brain penetration, although further understanding of the mechanism of LM10 is still required. Nonetheless, this could be in line with a study reporting that a non‐blood–brain barrier‐penetrating KMO inhibitor has beneficial effects on both peripheral and central phenotypes in an Alzheimer’s disease mouse model [24].

Notably, the PK studies were carried out with healthy mice. Rotenone treatment is reported to have a negative impact on the integrity of the blood–brain barrier [47], although contradicting data are also reported [48]. Therefore, it is possible that rotenone treatment in the efficacy model has resulted in a change of the blood‐to‐brain partition coefficients of the TDO inhibitors compared with those measured in the PK model.

There are several possible mechanisms through which TDO inhibition may ameliorate PD symptoms at the molecular or metabolic level. First of all, inhibition of TDO could lead to increased serotonin levels as a consequence of the increase in Trp, because Trp acts as a precursor in serotonin biosynthesis [49]. In the brains of PD patients, the concentration of serotonin is found to be decreased [50]. At neural synapses, increased serotonin levels as a consequence of TDO inhibition could have positive effects on cognitive function, thereby reducing depression [51], which is one of the most common nonmotor symptoms in PD with an average prevalence of 40% [52]. This concept was explored more than two decades ago by Madge and coworkers [53], who synthesized TDO inhibitors and combined TDO/5‐HT reuptake inhibitors [53]. Administration of these inhibitors to rats resulted in increased cerebrospinal Trp and serotonin levels [41]. Moreover, selective serotonin reuptake inhibitors (SSRIs) have been shown to significantly ameliorate depression in PD patients [54], while administration of an SSRI to rotenone‐treated rats resulted in improved motor function [55]. Secondly, TDO inhibition may decrease the levels of neurotoxic metabolites of Trp, more specifically, 3‐HK and quinolinic acid (QA) (Fig. 1). 3‐HK causes neuronal cell death by generating free radicals [56], while QA is a potent stimulant of the *N*‐methyl‐D‐aspartate (NMDA) receptor [57]. Intracerebroventricular injection of QA causes seizure activity in mice [58]. Recent studies indicate that QA may also have a direct effect on the formation of α‐synuclein inclusion bodies associated with PD. Tavassoly and coworkers showed that QA can form amyloid‐like fibrillar assemblies that can induce the formation of α‐synuclein aggregates in cells [59]. However, formation of these assemblies required high concentration of metabolite (at least 0.5 mg·mL^−1^, *i.e.*, 3 mM) and heating to high temperature (90 °C) [59]. Furthermore, the same investigators published that Trp can form similar structures, indicating that the effect is not specific for QA [59, 60].

The availability of human genetic evidence is an important aspect in the selection of new targets for therapeutic intervention. Such evidence, although lacking for TDO, is available for ACMSD, an enzyme further downstream in the kynurenine pathway (Fig. 1) [22, 23]. ACMSD catalyzes the formation of the neuroprotective picolinic acid, thereby reducing QA cytotoxicity (Fig. 1) [61]. Polymorphisms in the *ACMSD* locus are predicted to result in decreased ACMSD levels and increased QA levels [62]. Moreover, there is a report of an individual with a missense mutation in *ACMSD* and late‐onset PD [63]. Although the functional consequence of the mutation has not been characterized, computer‐assisted structural studies indicate that it impairs ACMSD activity [62]. Finally, a stop codon mutation in *ACMSD* is linked to an autosomal dominant neurological disease with epilepsy and features of parkinsonism, that is, familial cortical myoclonic tremor and epilepsy (FCMTE) [64]. While this is a relatively mild disorder with no evidence of neurodegeneration, it provides strong genetic evidence for the involvement of a deregulated kynurenine pathway in aberrant motor function and neurological disease.

In conclusion, our study shows for the first time in a relevant animal model that TDO inhibition with small molecule drugs may be beneficial for treatment of PD symptoms. Whereas levodopa acts only as a temporary relief of motor symptoms, our preclinical study indicates that administration of TDO inhibitors has beneficial effects on both motor and nonmotor symptoms of PD. The precise mechanism underlying the disease‐modifying effects of TDO inhibition and the relative contribution of peripheral *versus* brain TDO require further study.

## Materials and Methods

### Compound library and TDO inhibitors

Human TDO was screened at the Pivot Park Screening Centre (PPSC) (Oss, The Netherlands) (www.ppscreeningcentre.com) using the NFK Green™ assay technology developed at Netherlands Translational Research Center B.V. (NTRC) (www.residencetimer.com) [31]. The PPSC library consisted of a diverse set of 87,000 lead‐like compounds purchased at Specs (Delft, The Netherlands). NTRC 3531‐0 is derived from a TDO inhibitor identified in this screen with a unique 3‐phenyl‐1*H*‐indole scaffold (Fig. 2A). The synthesis of NTRC 3531‐0 is described in patent application WO2018/011227 A1 [32]. The compound is available for academic research under a Materials Transfer Agreement with NTRC. LM10 was synthesized according to the protocol described by Dolušić and coworkers [29]. The IDO1 inhibitor epacadostat, used as a reference inhibitor in the biochemical and cell‐based assays for IDO1, was purchased at MedKoo (cat. no. 206461). Before testing in biochemical and cell‐based assays, compounds were dissolved in dimethylsulfoxide (DMSO). For dose‐response testing, the compound stock was diluted in √10‐fold steps in DMSO to obtain a 10‐point dilution series, followed by further dilution in aqueous buffers, as specified below for the different assays.

### TDO biochemical assay

Recombinant TDO was purified and its biochemical activity was measured with the NFK Green assay, as described [31]. In short, compounds were diluted in DMSO and finally in TDO reaction buffer, consisting of 100 mm NaH_2_PO_4_, pH 7.0, supplemented with 0.01% Tween‐20 (Sigma, cat. no. P7949). hTDO enzyme was diluted in reaction buffer supplemented with ascorbic acid, as described [31], and combined with inhibitor solution in a black 384‐well plate (Corning, cat. no. 3537). The final concentration of hTDO in the assay was 50 nm. After incubation for 60 min at room temperature, the enzymatic reaction was initiated by the addition of Trp to a final concentration of 200 µm. The reaction was stopped after incubation for 15 min at room temperature by the addition of NFK Green. Plates were read on an EnVision multimode reader (Perkin Elmer, Waltham, MA, USA). For high‐throughput screening, the assay was further miniaturized to 1536‐well format and the compound library was screened at a single concentration of 10 µm. For determination of IC_50_ values, the effect of inhibitors was determined in 10‐point duplicate dose‐response curves. Graphs were fitted to a four‐parameter logistics equation in XLfit (ID Business Solutions, Ltd., Guildford, UK) from which IC_50_ values were calculated. The same assay was performed for mTDO (final concentration of 100 nm), except that the preincubation with compound was performed for 30 min and, after addition of Trp to a final concentration of 400 µm, the enzymatic reaction was allowed to proceed for 60 min.

### IDO1 biochemical assay

Activity on recombinant hIDO1 was determined with the NFK Green assay as described above for TDO [31], except that the reaction buffer consisted of 50 mm NaH_2_PO_4_, pH 7.0, supplemented with 0.01% Tween‐20 and 1% glycerol. In addition to ascorbic acid (final concentration of 10 mm), the enzyme solution contained catalase (10 µg·mL^−1^) and methylene blue (10 µm). The final hIDO1 enzyme concentration was 25 nm, and the final Trp concentration was 100 µm [31]. The enzyme was preincubated with compound for 30 min, after which the enzymatic reaction with Trp was allowed to proceed for 60 min.

### Cell lines and culture

The GripTite™ HEK‐293 MSR cell line was purchased from Thermo Fisher (cat. no. R79507; Waltham, MA, USA), the human colorectal carcinoma SW48 cell line (cat. no. CCL‐231) from the American Type Culture Collection (ATCC) (Manassas, VA, USA), and the mouse glioma cell line GL‐261 (cat. no. ACC 802) from the Deutsche Sammlung von Mikroorganismen und Zellkulturen (DSMZ) (Braunschweig, Germany). HEK‐hTDO and HEK‐hIDO1 cells were cultured in DMEM supplemented with 10% fetal bovine serum (FBS), 1% Penicillin/Streptomycin (P/S) solution, 1% MEM nonessential amino acids, 600 μg·mL^−1^ geneticin and 10 μg·mL^−1^ blasticidin. SW48 cells were cultured in RPMI 1640 supplemented with 10% bovine calf serum (BCS) and 1% P/S. GL‐261‐mTDO cells were cultured in DMEM supplemented with 10% BCS, 1% P/S and 10 μg·mL^−1^ blasticidin. DMEM medium contains 78 µm Trp and RPMI 1640 contains 24.5 µm Trp.

## Generation of *TDO2‐* and *IDO1*‐overexpressing cell lines

Full‐length *hTDO2*, *mTDO2,* and *hIDO1* cDNAs were synthesized and cloned in the expression vector pEF6v5 (Thermo Fisher, cat. no. V96120), harboring an ampicillin and blasticidin resistance marker gene, at BaseClear (Leiden, The Netherlands). Using these vectors, cell lines expressing *hTDO2* or *hIDO1* were generated by transfection of GripTite™ HEK‐293 MSR cells. A cell line expressing *mTDO2* was generated by transfection of GL‐261 cells. HEK‐293 MSR and GL‐216 cells lack endogenous expression of *TDO2* and *IDO1* as determined by quantitative real‐time PCR (qPCR). The absence of TDO and IDO1 in HEK‐293 MSR cells was further confirmed by immunoblot analysis. Cells were transfected with Lipofectamine 3000 (Thermo Fisher). Single cell clones were selected with blasticidin selection and limited dilution in 384‐well culture plates. Trp‐converting activity in the clones was tested with NFK Green or 4‐dimethylaminobenzaldehyde (pDMAB) [65]. To assess the stability of *TDO2* or *IDO1* expression, clones were cultured for four weeks in the absence of blasticidin, during which they were tested weekly for maintenance of the Trp‐catabolizing activity. Afterward, the gene expression levels, the protein expression levels and the inhibitory activity of reference compounds were determined in the resulting sublines. Cell‐based assays with the transfected cell lines were performed in the absence of blasticidin.

### Gene expression analysis

Expression of *TDO2* and *IDO1* in human and mouse cell lines transfected with cDNA expression vectors was confirmed by qPCR, following standard protocols [66]. Sequences of DNA oligonucleotide primers used are provided in Table S1. Expression of *TDO2* and *IDO1* was normalized using the expression of housekeeping genes. For the analysis of *hTDO2* and *hIDO1* gene expression, this was the gene for β‐actin (*ACTB*). For the analysis of the expression of *mTDO2* or *mIDO1*, the control genes were mouse *ACTB* and glyceraldehyde‐3‐phosphate dehydrogenase (*GAPDH*).

### Immunoblot analysis

The expression of hTDO and hIDO1 in cell lines was also confirmed by SDS‐polyacrylamide gel electrophoresis (SDS/PAGE) and immunoblot analysis. hTDO was detected with mouse polyclonal anti‐TDO antibody (Abcam, cat. no. ab76859; Cambridge, UK), horseradish peroxidase (HRP) conjugated anti‐mouse IgG (Cell Signaling, cat. no. 7076S; Danvers, MA, USA), and enhanced chemiluminescence (ECL) using Clarity™ Western ECL substrate (Bio‐Rad, cat. no. 170‐5060; Hercules, CA, USA). hIDO1 was detected with anti‐IDO1 rabbit monoclonal antibody (Cell Signaling, cat. no. 86630), HRP‐conjugated anti‐rabbit IgG (Cell Signaling, cat. no. 7074) and ECL. To control for equal loading of the samples on SDS/PAGE gels, the blots were stripped using Restore™ Plus Western Blot Stripping Buffer (Thermo Fisher, cat. no. 46430) after which β‐actin was detected using rabbit polyclonal anti‐β‐actin antibody (Cell Signaling, cat. no. 4967), HRP‐conjugated anti‐rabbit IgG (Cell Signaling) and ECL.

### Cell‐based assays for TDO and IDO1

Inhibition of Trp‐to‐NFK conversion in HEK‐hTDO, HEK‐hIDO1, and SW48 cells was measured with NFK Green, essentially as described previously for cancer cell lines [31]. Cell density, incubation time, and Trp concentrations were optimized. For the TDO assay with HEK‐hTDO and SW48 cells, 200 µm Trp was added to the culture medium. No additional Trp was added for the IDO1 assay with HEK‐hIDO1 cells. The incubation time with compound was 42 h for the assays with HEK‐hTDO and HEK‐hIDO1 cells, and 18 h for the assay with SW48 cells.

Inhibition of Trp‐metabolizing activity in GL‐261‐mTDO cells was measured with pDMAB [65]. The cells were incubated with compound for 1 h, after which 200 µm Trp was added. After incubation for 42 h, 5% trichloroacetic acid (Fisher) in Milli‐Q was added to each well and incubated for 1 h at 55 °C to hydrolyze NFK to Kyn. The plates were centrifuged for 10 min at 2900 *g*. The supernatant was transferred to another plate and 2% pDMAB (Fisher Chemicals, Waltham, MA, USA) dissolved in acetic acid (Acros) was added. After 10 min incubation at room temperature, the absorbance at 480 nm was measured to determine the production of Kyn.

In parallel with the cell‐based NFK Green assays, the cytotoxicity of the compounds was determined in cell viability assays using ATPlite™ 1Step (PerkinElmer, Groningen, The Netherlands) [67]. NTRC 3531‐0 and LM10 were not cytotoxic to any cell line used in this study.

### Pharmacokinetic analysis, brain penetration, and quantification of Trp and Kyn

*In vivo* half‐life and oral bioavailability of NTRC 3531‐0 and LM10 were determined following single p.o. and i.v. administration to male CB57BL/6 mice at Aurigon (Dunakeszi, Hungary). In addition, plasma and brain levels after repeated dosing were determined by treating mice for five consecutive days p.o. daily (QD). Nine mice were used per dosing group. Plasma and brain samples were collected from three mice per dosing group at each time point. For plasma sampling, this enabled sampling at three time points (including pretreatment) from each mice. Formulations were prepared in DMSO, Kolliphor^®^ EL, 5% D‐mannitol in mineral water at a volume ratio of 1:1:8. Mice treated with a single dose of compound (i.v. or p.o.) and vehicle‐treated mice were fasted for approximately 4 h prior to dosing, and regained access to feed 2 h after dosing. Mice treated for 5 days had free access to feed. Blood samples were collected in K3‐EDTA tubes from the retro‐orbital plexus of the mice at 0.083, 0.25, 0.5, 1, 2, 7, 12, and 24 h after i.v. or p.o. dosing. Plasma samples were obtained by cooling of the blood samples in ice‐water followed by centrifugation at 2000 g for 10 min at 4 °C, and collection of the supernatant, which were immediately frozen on dry‐ice. Brains were collected immediately after the blood sampling at 1, 12, and 24 h after single and repeated p.o. dosing, after exsanguination of the animals to the standard extent, and were snap frozen in liquid nitrogen. Pharmacokinetic parameters (*C*_max_, *t*_1/2_ and AUC) were determined by LC‐MS/MS analysis of the plasma samples after acetonitrile extraction. To determine brain penetration, compound levels were determined in homogenates prepared from the brains. The brain‐to‐plasma partition coefficient (Kp) was determined as AUC_brain_/AUC_plasma_. The unbound brain‐to‐plasma partition coefficient (Kp_uu_) was determined as (AUC_brain_ × unbound fraction in brain tissue)/(AUC_plasma_ × unbound fraction in plasma). Quantification of Trp and Kyn levels was additionally performed by LC‐MS/MS in both the plasma samples and brain homogenates. The exact timepoints of blood sampling for these mice are listed in Table S2.

### Rotenone model, surgery, and administration of inhibitors

Seven‐week‐old male C57BL/6NCrl mice (Charles River, The Netherlands) were housed at room temperature under 12 h light/dark cycle. Animal procedures were approved by the Ethical Committee of Animal Research of Utrecht University, Utrecht, The Netherlands. Food and water was provided *ad libitum*. Mice underwent stereotaxic surgery under isoflurane anesthesia: A hole was drilled in the skull, a cannula inserted in the right striatum and 5.4 µg of freshly prepared rotenone (dissolved in DMSO) was infused. The following stereotaxic coordinates were used: AP + 0.4, ML −2.0 from bregma and DV −3.3 below dura.

Two independent experiments were performed involving two cohorts of mice, with ten mice per dosing or control group in total. To control for the effect of rotenone treatment, sham‐operated mice were injected with vehicle. TDO inhibitors were administered from seven days after surgery, once daily by oral gavage. Formulations were prepared in 5% DMSO, 5% Kolliphor^®^ EL, 5% D‐mannitol in water. Both inhibitors were administrated at three different doses: NTRC 3531‐0 at 25 mg·kg^−1^ (D1), 50 mg·kg^−1^ (D2) and 100 mg·kg^−1^ (D3), and LM10 at 12.5 mg·kg^−1^ (D1), 25 mg·kg^−1^ (D2) and 50 mg·kg^−1^ (D3). The highest dose of NTRC 3531‐0 (*i.e.*, 100 mg·kg^−1^) was predicted to result in at least 90% TDO inhibition, lasting for approximately 12 hours, based on the PK properties of NTRC 3531‐0 after p.o. administration, and an IC_90_ of 2.5 µM in the cell‐based HEK‐hTDO assay (Fig. 3B). The effect of inhibitor treatment was determined by comparison to vehicle‐treated mice. For ethical reasons it was not possible to dose the TDO inhibitors p.o. twice daily. Forty‐two days after surgery, mice were euthanized by decapitation.

### Motor symptoms assessment

Motor function was assessed in the rotarod test by placing mice on an accelerating rod with speeds starting at 2 rpm and gradually increasing to 20 rpm. The latency to fall was recorded for a maximum of 300 s. Baseline motor function was tested seven days after surgery, and subsequently, motor function was tested every seven days until day 42.

### Spatial memory test

The spatial memory test measures the ability of the mice to react to a spatial novelty [36]. Mice were individually submitted to seven consecutive sessions of six minutes. During the first session, mice were placed in an empty cage. During the 2^nd^ to 4^th^ session, five objects were present and mice were placed into the cage to habituate to the configuration of the objects (habituation phase). During an interval of three minutes between two sessions, the animals were returned to a waiting cage. Before the 5^th^ session, the spatial test session, the configuration of the objects was changed by moving two objects (displaced objects, DO) and leaving the other three objects at the same position (nondisplaced objects, NDO). In all sessions, the total activity of the animal was measured. From session 2 to session 5, object exploration was evaluated on the basis of the mean time spent by the animal in contact with the different objects. The ability of the animals to selectively react to the spatial change was analyzed by calculating the spatial re‐exploration index (DO[S5] – DO[S4] = DO and NDO[S5] – NDO[S4] = NDO). The time the animals interacted with the DO minus the time they interact with the NDO was used for the analysis. The raw data of the spatial memory test can be found in Tables S3–S6.

### Intestinal transit and colon length

Thirty minutes before sacrificing the mice, 2.5% (v/v) Evans blue solution in 1.5% methylcellulose was administered intragastrically (0.3 mL per animal). Intestinal transit was measured as the distance from the pylorus to the most distal point of migration of the Evans blue dye. In addition, the length of the colon was determined as a sign of intestinal inflammation [28].

### iDISCO

The number of TH‐positive cells and the morphology of microglia (Iba1 immunolabeling) in the brains of mice was determined by immunolabeling‐enabled three‐dimensional imaging of solvent‐cleared organs (iDISCO) [37]. Brains were cut in hemispheres and dehydrated with a methanol/H_2_O series: 20%, 40%, 60%, 80%, 100% and 100% for 1 h each. Samples were incubated overnight in 66% DCM/33% methanol at room temperature, with shaking. The next day, the samples were washed twice in 100% methanol at room temperature, and bleached in chilled fresh 5% H_2_O_2_ in methanol overnight at 4 °C. The samples were rehydrated with a methanol/H_2_O series: 80%, 60%, 40%, 20% and phosphate‐buffered saline (PBS) or 1 h each at room temperature. They were then washed twice in PTx.2 (PBS with 0.2% Triton X‐100) at room temperature for 1 h.

For immunolabeling, samples were incubated in permeabilization buffer (400 mL PTx.2 supplemented with 11.5 g glycine and 100 mL of DMSO) for 2 days at 37 °C. After permeabilization, samples were blocked in blocking solution for 2 days at 37 °C. Primary antibodies (sheep anti‐TH, Pel freez, cat. no. P60101; rabbit anti‐Iba1, Thermo Fisher, cat. no. PA5‐27436) diluted in PTwH/5% DMSO/3% donkey serum (PTwH being defined as PBS with 0.2% Triton X‐100 and 10 μg mL^−1^ heparin) were then incubated with the tissue for 5 days at 37 °C. After incubation, samples were washed in PTwH for 4 to 5 times until the next day. Secondary antibodies (Alexa Fluor® 647‐conjugated donkey anti‐sheep and Alexa Fluor® 594‐conjugated donkey anti‐rabbit) were diluted in PTwH with 3% donkey serum and incubated with the samples for 5 days at 37 °C. Samples were washed with PTwH for 4 to 5 times until the next day.

Samples were dehydrated in a methanol/H_2_O series: 20%, 40%, 60%, 80%, 100%, and 100% for 1 h each. The hemispheres were subsequently put in 66% DCM/33% methanol for 3 h at room temperature. They were incubated twice in 100% dichloromethane for 15 min to wash away the methanol. Finally, samples were incubated in dibenzyl ether overnight. The tube was completely filled to prevent any oxidization.

### Light sheet imaging

Samples were imaged in horizontal orientation with an UltraMicroscope II (LaVision BioTec, Bielefeld, Germany) light sheet microscope equipped with Imspector software (LaVision BioTec, version 5.0285.0). Images were taken with a Neo sCMOS camera (Andor) (2560 × 2160 pixels; pixel size: 6.5 × 6.5 μm^2^). Samples were scanned with double‐sided illumination, a sheet NA of 0.148348 and a step‐size of 2.5 μm and 0.5 μm using the horizontal focusing light sheet scanning method with the optimal amount of steps and using the contrast blending algorithm. The effective magnification for all images was 1.36x (zoombody × objective + dipping lens = 0.63 × 2.152). A Coherent OBIS 647–120 LX with 676/29 filter and LS 561‐100 laser was used. After image acquisition, full‐stained 3D brain images were obtained. TH‐immunopositive neurons and Iba1‐positive cells in the SN were analyzed using Bitplane Imaris 9.0. To measure TH‐immunopositive neurons, the automatic ‘spots’ measurement function was used with a median diameter of 16.3 μm. For the Iba1‐positive cells, the ‘Imaris Surfaces’ tool was used for measurement of the volume and the space occupied by these cells in the SN.

### Immunofluorescent microscopy

Expression of GFAP and α‐synuclein in colonic tissue was determined using confocal microscopy (Leica SP8). The colons of the mice were embedded in paraffin. Sections of 15 µm were cut and incubated with 0.3% (v/v) H_2_O_2_ for 30 min, rehydrated and incubated with citrate buffer. Following blocking with serum, the sections were incubated overnight with rabbit anti‐α‐synuclein (Millipore, cat. no. AB5038; Burlington, MA, USA) at a dilution of 1 : 1000, or rabbit anti‐GFAP (Dako, cat. no. Z0334), 1 : 1000. After incubation with Alexa® 488‐labeled donkey anti‐rabbit secondary antibody, the slides were mounted using Vectashield^®^ medium for fluorescence with DAPI (Vector Laboratories, cat. no. H‐1000; Burlingame, CA, USA).

Immunofluorescence images were made using a Keyence BZ‐9000 microscope. GFAP and α‐synuclein staining were quantified by calculation of the corrected total fluorescence (CTF) with the formula: integrated density – (area × mean fluorescence of background reading).

### Statistical analyses

Experimental results of the PK study and the rotenone model are expressed as mean ± SEM. Differences in metabolite levels between the groups of the PK study were analyzed with a two‐way ANOVA, followed by Dunnett’s multiple comparison test. The effect of treatment with inhibitor was related to the effect of vehicle treatment. Differences between groups of the rotenone model were analyzed with a two‐way ANOVA, followed by a Tukey's multiple comparison test. The effect of injection with rotenone (rotenone/vehicle) was related to the effect of the sham surgery (sham/vehicle). Furthermore, the effect of treatment with inhibitor (rotenone/TDO inhibitor) was related to the effect of vehicle treatment (rotenone/vehicle). For the rotarod test, data were analyzed with a general linear model repeated measures ANOVA with the within‐subjects factor ‘time’ and the between‐subjects factors ‘surgery’ and ‘TDO inhibitor treatment’. Results are considered statistically significant when *P* < 0.05. The individual *P* values of the multiple comparisons presented in the bar charts are listed in Table S7. Analyses were performed using spss (version 22.0; IMB Corp., Armonk, NY, USA) and graphpad prism (version 6.07; GraphPad Software, San Diego, CA, USA).

## Conflict of interest

R.C. Buijsman and G.J.R. Zaman are managing directors and shareholders of Netherlands Translational Research Center B.V. The other authors have no potential conflicts of interest.

## Author contributions

PP, RCB, GJRZ, and ADK conceived and designed the experiments. PP, YG, NW, MT, MM, DV, AMD, FC, JW, JGS, and JM performed the experiments. PP, YG, NW, MM, DV, AMD, JCMU, JM, RCB, GJRZ, and ADK analyzed the data. YA and RJP provided facilities. RCB, GJRZ, and ADK supervised the study. PP, YG, and GJRZ wrote the paper.

### Peer Review

The peer review history for this article is available at https://publons.com/publon/10.1111/febs.15721.

## Supporting information

**Table S1.** DNA sequences of oligonucleotide primers used for quantitative real‐time PCR analysis of *TDO2* and *IDO1* gene expression in cell lines.
**Table S2.** Time (hh:mm) of treatment and blood sampling of the mice analyzed for plasma Trp and Kyn levels in Figure 4.
**Table S3.** Raw data of the spatial memory test at day 7.
**Table S4.** Raw data of the spatial memory test at day 14.
**Table S5.** Raw data of the spatial memory test at day 28.
**Table S6.** Raw data of the spatial memory test at day 42.
**Table S7.** Overview of the *p* values of the multiple comparisons presented in the denoted bar charts.
