# Supplementary material for: Pharmacological validation of TDO as a target for Parkinson’s disease
Source: FEBS J. 2021 Feb 18;288(14):4311–31. doi: 10.1111/febs.15721 (PMC8359396; doi:10.1111/febs.15721)
Supplement: Supplementary file 1 — Table S1. DNA sequences of oligonucleotide primers used for quantitative real‐time PCR analysis of TDO2 and IDO1 gene expression in cell lines. Table S2. Time (hh:mm) of treatment and blood sampling of the mice analyzed for plasma Trp and Kyn levels in Figure 4. Table S3. Raw data of the spatial memory test at day 7. Table S4. Raw data of the spatial memory test at day 14. Table S5. Raw data of the spatial memory test at day 28. Table S6. Raw data of the spatial memory test at day 42. Table S7. Overview of the p values of the multiple comparisons presented in the denoted bar charts. [file FEBS-288-4311-s001.zip › febs15721-sup-0001-TableS1-S7.pdf]

FEBS PRESS
science publishing by scientists

WILEY

The FEBS Journal

www.febsjournal.org

Pharmacological validation of TDO as a target for Parkinson's disease

Paula Perez-Pardo, Yvonne Grobben, Nicole Willemsen-Seegers, Mitch Hartog, Michaela Tutone, Michelle Muller, Youri Adolfs, Ronald Jeroen Pasterkamp, Diep Vu-Pham, Antoon M. van Doornmalen, Freek van Cauter, Joeri de Wit, Jan Gerard Sterrenburg, Joost C.M.Uitdehaag, Jos de Man, Rogier C. Buijsman, Guido J.R. Zaman and Aletta D. Kraneveld

DOI: 10.1111/febs.15721

Supplementary Data

Table S1. DNA sequences of oligonucleotide primers used for quantitative real-time PCR analysis of *TDO2* and *IDO1* gene expression in cell lines.

Gene	Species	Direction	Sequence
TDO2	human	forward	CTTAGTAAAGGTGAAAGACGG
	human	reverse	GTCCATAAGAGAAGTCAGCA
TDO2	mouse	forward	ATGAGTGGGTGCCCGTTT
	mouse	reverse	GGCTCTGTTTACACCAGTTTGAG
IDO1	human	forward	TTAGAGTCAAATCCCTCAGTCC
	human	reverse	TGCAGATGGTAGCTCCTC
IDO1	mouse	forward	GGCTTTGCTCTACCACATCCAC
	mouse	reverse	TAGCCACAAGGACCCAGGG
ACTB	human	forward	CAAGAGATGGCCACGGCTGCTTCCA
	human	reverse	GCATGGAGTTGAAGGTAGTTTCG
ACTB	mouse	forward	GGCTGTATTCCCCTCCATCG
	mouse	reverse	CCAGTTGGTAACAATGCCATGT
RPS18	human	forward	GACAACAAGCTCCGTGAAGA
	human	reverse	AGAAGTGACGCAGCCCTCTA
GAPDH	mouse	forward	ACGGATTTGGTCGTATTGGG
	mouse	reverse	CGCTCCTGGAAGATGGTGAT
RPL37	mouse	forward	TCTGTGGCAAGACCAAGATG
	mouse	reverse	GACAGCAGGGCTTCTACTGG

Table S2. Time (hh:mm) of treatment and blood sampling of the mice analyzed for plasma Trp and Kyn levels in Figure 4. The treatment and sampling within the subgroups of three mice (indicated by 1-3, 4-6 and 7-9) was performed with three-minute intervals, of which the time of the first treatment or sampling per subgroup is listed in the table.

	**Vehicle**			**NTRC 3531-0**			**LM10**		
**Mouse**	**1-3**	**4-6**	**7-9**	**1-3**	**4-6**	**7-9**	**1-3**	**4-6**	**7-9**
Treatment	11:15	10:00	10:18	10:00	09:15	10:20	11:06	10:09	10:57
5 min	11:20			10:05			11:11		
15 min		10:15				10:35		10:24	
30 min			10:48		09:45				11:27
1 h	12:15			11:00			12:06		
2 h			12:18			12:20			12:57
7 h		17:00			16:15			17:09	
12 h		22:00			21:15			22:09	
24 h			10:18			10:25			10:57

Table S3. Raw data of the spatial memory test at day 7. The data are reported as the time the animals interacted with the displaced objects (DO; calculated as DO[S5] – DO[S4]), the time they interacted with the non-displaced objects (NDO; calculated as NDO[S5] – NDO[S4]), and the difference between DO and NDO. The data reported for DO – NDO is used for the statistical analysis and is shown in Figure 5C.

	Sham			Rotenone						
	**Vehicle**	**NTRC D3**	**LM10 D3**	**Vehicle**	**NTRC D1**	**NTRC D2**	**NTRC D3**	**LM10 D1**	**LM10 D2**	**LM10 D3**
DO	29	13	18	23	14	15	10	20	19	13
	15	23	20	18	19	25	24	7	14	19
	13	23	20	19	15	26	17	18	20	18
	19	19	20	18	14	13	15	18	19	10
	15	19	17	17	24	9	19	15	18	17
	13	12	9	16	19	23	10	21	14	22
	21	24	23	17	12	13	17	12	10	14
	15	19	15	19	16	14	15	18	20	16
	17	20	21	18	19	19	9	23	21	24
	10	15	15	17	20	16	11	13	10	19
NDO	9	7	9	7	6	5	2	4	7	5
	4	3	5	10	8	11	9	1	6	5
	4	11	7	6	4	6	6	10	6	4
	3	5	4	4	6	3	6	3	5	7
	4	5	4	8	6	3	4	8	7	5
	7	3	3	3	3	12	3	6	7	7
	7	9	6	4	4	4	2	4	1	5
	5	8	3	10	7	5	2	6	2	4
	9	3	6	3	4	5	3	7	9	9
	2	3	6	9	9	8	6	6	3	11
DO – NDO	20	6	9	16	8	10	8	16	12	8
	11	20	15	8	11	14	15	6	8	14
	9	12	13	13	11	20	11	8	14	14
	16	14	16	14	8	10	9	15	14	3
	11	14	13	9	18	6	15	7	11	12
	6	9	6	13	16	11	7	15	7	15
	14	15	17	13	8	9	15	8	9	9
	10	11	12	9	9	9	13	12	18	12
	18	17	15	15	15	14	6	16	12	15
	8	12	9	8	11	8	5	7	7	8

Table S4. Raw data of the spatial memory test at day 14. The data are reported as the time the animals interacted with the displaced objects (DO; calculated as DO[S5] – DO[S4]), the time they interacted with the non-displaced objects (NDO; calculated as NDO[S5] – NDO[S4]), and the difference between DO and NDO. The data reported for DO – NDO is used for the statistical analysis and is shown in Figure 5C.

	**Sham**			**Rotenone**						
	**Vehicle**	**NTRC D3**	**LM10 D3**	**Vehicle**	**NTRC D1**	**NTRC D2**	**NTRC D3**	**LM10 D1**	**LM10 D2**	**LM10 D3**
DO	21	13	13	15	20	20	17	21	15	21
	9	14	13	20	20	13	21	21	19	27
	17	13	21	14	20	19	10	15	21	18
	23	18	16	25	10	16	7	12	14	14
	16	15	10	19	14	19	18	11	12	12
	19	10	14	16	13	20	15	20	16	17
	19	11	16	15	11	12	12	16	18	18
	14	22	20	17	17	12	13	13	17	11
	18	17	17	20	19	21	17	16	17	17
	15	24	15	22	16	16	16	20	15	18
NDO	3	5	7	8	7	6	2	10	4	10
	3	3	7	3	4	5	6	5	3	11
	10	7	2	10	10	6	5	1	7	4
	4	6	5	4	3	8	4	3	5	5
	5	5	2	8	6	4	8	6	7	7
	7	1	4	5	5	6	3	8	4	5
	9	1	9	3	4	4	2	6	8	8
	7	10	8	9	5	4	2	5	9	3
	5	9	6	8	8	7	6	5	6	6
	4	12	4	10	5	6	8	8	3	6
DO – NDO	18	8	6	6	13	14	15	11	11	11
	6	11	6	17	16	8	15	16	16	16
	7	6	19	4	10	13	5	14	14	14
	19	12	11	21	7	8	3	9	9	9
	11	10	8	11	8	15	10	5	5	5
	12	9	10	11	8	14	12	12	12	12
	10	10	7	12	7	8	10	10	10	10
	7	12	12	8	12	8	11	8	8	8
	13	8	11	12	11	12	11	11	11	11
	11	12	11	12	11	10	8	12	12	12

Table S5. Raw data of the spatial memory test at day 28. The data are reported as the time the animals interacted with the displaced objects (DO; calculated as DO[S5] – DO[S4]), the time they interacted with the non-displaced objects (NDO; calculated as NDO[S5] – NDO[S4]), and the difference between DO and NDO. The data reported for DO – NDO is used for the statistical analysis and is shown in Figure 5C.

	**Sham**			**Rotenone**						
	**Vehicle**	**NTRC D3**	**LM10 D3**	**Vehicle**	**NTRC D1**	**NTRC D2**	**NTRC D3**	**LM10 D1**	**LM10 D2**	**LM10 D3**
DO	15	16	19	18	23	17	14	15	18	16
	15	24	16	11	16	16	18	14	18	13
	21	8	14	12	20	14	14	19	20	16
	10	15	16	15	18	20	12	23	17	25
	12	21	18	20	23	20	11	16	21	11
	20	24	16	22	21	10	22	16	19	12
	18	23	13	18	11	17	16	10	14	20
	17	10	10	18	21	16	14	17	21	13
	19	13	14	11	19	22	14	20	14	22
	18	18	16	12	25	19	10	19	15	15
NDO	6	4	8	7	6	8	3	9	3	6
	3	4	3	9	10	7	6	4	9	3
	3	2	9	7	6	5	8	6	6	10
	5	8	6	1	4	3	1	7	10	7
	6	4	10	4	4	7	2	6	6	5
	9	9	1	5	6	3	9	8	6	4
	10	6	6	5	3	7	6	2	3	8
	5	3	3	5	9	5	6	2	7	5
	7	7	6	5	3	7	5	6	5	6
	9	5	6	3	6	4	1	9	2	9
DO – NDO	9	12	11	11	17	9	11	6	15	10
	12	20	13	3	6	9	12	10	9	10
	18	6	5	5	14	9	6	13	14	6
	5	7	10	14	14	17	11	16	7	18
	6	17	8	16	19	13	9	10	15	6
	11	15	15	17	15	7	13	8	13	8
	8	17	7	13	8	10	10	8	11	12
	12	7	7	13	12	11	8	15	14	8
	12	6	8	6	16	15	9	14	9	16
	9	13	10	9	19	15	9	10	13	6

Table S6. Raw data of the spatial memory test at day 42. The data are reported as the time the animals interacted with the displaced objects (DO; calculated as DO[S5] – DO[S4]), the time they interacted with the non-displaced objects (NDO; calculated as NDO[S5] – NDO[S4]), and the difference between DO and NDO. The data reported for DO – NDO is used for the statistical analysis and is shown in Figure 5C.

	**Sham**			**Rotenone**						
	**Vehicle**	**NTRC D3**	**LM10 D3**	**Vehicle**	**NTRC D1**	**NTRC D2**	**NTRC D3**	**LM10 D1**	**LM10 D2**	**LM10 D3**
DO	13	18	18	4	0	4	12	8	8	20
	18	15	14	8	3	3	25	10	9	9
	16	16	17	10	10	9	11	7	6	16
	18	15	14	5	5	3	14	6	1	6
	12	14	21	2	3	2	14	9	2	13
	20	18	18	4	4	4	13	4	10	15
	19	16	18	4	6	7	15	5	11	12
	21	11	13	2	3	9	14	4	7	13
	15	16	15	6	2	6	14	6	4	12
	14	17	20	3	6	5	16	2	7	15
NDO	5	0	4	3	1	1	8	4	2	7
	2	2	0	4	0	0	9	6	5	5
	3	3	8	8	8	5	2	3	1	5
	7	4	1	2	4	2	5	5	0	2
	5	6	1	3	2	2	1	7	0	4
	10	2	2	2	4	2	5	1	6	7
	5	1	6	2	4	3	2	0	6	4
	10	1	2	0	1	7	4	2	5	7
	2	7	3	3	0	3	6	4	3	1
	7	3	2	1	0	4	5	1	4	6
DO – NDO	8	18	14	1	-1	3	4	4	6	13
	16	13	14	4	3	3	16	4	4	4
	13	13	9	2	2	4	9	4	5	11
	11	11	13	3	1	1	9	1	1	4
	7	8	20	-1	1	0	13	2	2	9
	10	16	16	2	0	2	8	3	4	8
	14	15	12	2	2	4	13	5	5	8
	11	10	11	2	2	2	10	2	2	6
	13	9	12	3	2	3	8	2	1	11
	7	14	18	2	0	1	11	1	3	9

Table S7. Overview of the *p* values of the multiple comparisons presented in the denoted bar charts.

		Sham			Rotenone						
		Vehicle	NTRC D3	LM10 D3	Vehicle	NTRC D1	NTRC D2	NTRC D3	LM10 D1	LM10 D2	LM10 D3
Spatial memory at day 42 (Fig. 5C)											
Sham	Vehicle		n.s.	n.s.	< 0.0001	< 0.0001	< 0.0001	n.s.	< 0.0001	< 0.0001	n.s.
	NTRC D3			n.s.	< 0.0001	< 0.0001	< 0.0001	n.s.	< 0.0001	< 0.0001	n.s.
	LM10 D3				< 0.0001	< 0.0001	< 0.0001	n.s.	< 0.0001	< 0.0001	n.s.
Rotenone	Vehicle					n.s.	n.s.	< 0.001	n.s.	n.s.	n.s.
	NTRC D1						n.s.	< 0.001	n.s.	n.s.	n.s.
	NTRC D2							< 0.001	n.s.	n.s.	n.s.
	NTRC D3								< 0.0001	< 0.0001	n.s.
	LM10 D1									n.s.	n.s.
	LM10 D2										n.s.
	LM10 D3										
TH-positive cells (Fig. 6A)											
Sham	Vehicle		n.s.	n.s.	< 0.0001	< 0.0001	< 0.0001	< 0.0001	< 0.0001	< 0.0001	< 0.0001
	NTRC D3			n.s.	< 0.0001	< 0.0001	< 0.0001	< 0.0001	< 0.0001	< 0.0001	< 0.0001
	LM10 D3				< 0.0001	< 0.0001	< 0.0001	< 0.0001	< 0.0001	< 0.0001	< 0.0001
Rotenone	Vehicle					n.s.	n.s.	< 0.01	n.s.	< 0.01	< 0.01
	NTRC D1						n.s.	< 0.05	n.s.	< 0.01	< 0.01
	NTRC D2							< 0.05	n.s.	< 0.01	< 0.01
	NTRC D3								< 0.01	< 0.01	< 0.001
	LM10 D1									< 0.001	< 0.001
	LM10 D2										n.s.
	LM10 D3										
Microglial cell volume (Fig. 6C)											
Sham	Vehicle		n.s.	n.s.	< 0.05	< 0.05	< 0.05	< 0.05	< 0.05	< 0.05	< 0.05
	NTRC D3			n.s.	< 0.05	< 0.05	< 0.05	< 0.05	< 0.05	< 0.05	< 0.05
	LM10 D3				< 0.05	< 0.05	< 0.05	< 0.05	< 0.05	< 0.05	< 0.05
Rotenone	Vehicle					n.s.	n.s.	< 0.05	n.s.	< 0.05	< 0.05
	NTRC D1						n.s.	< 0.05	n.s.	n.s.	< 0.05
	NTRC D2							< 0.05	n.s.	n.s.	< 0.05
	NTRC D3								< 0.05	< 0.05	n.s.
	LM10 D1									n.s.	< 0.05
	LM10 D2										< 0.05
	LM10 D3										
Microglial occupancy index (Fig. 6D)											
Sham	Vehicle		n.s.	n.s.	< 0.05	n.s.	n.s.	n.s.	n.s.	n.s.	n.s.
	NTRC D3			n.s.	< 0.05	n.s.	n.s.	n.s.	n.s.	n.s.	n.s.
	LM10 D3				< 0.05	n.s.	n.s.	n.s.	n.s.	n.s.	n.s.
Rotenone	Vehicle					n.s.	n.s.	n.s.	n.s.	n.s.	n.s.
	NTRC D1						n.s.	n.s.	n.s.	n.s.	n.s.
	NTRC D2							n.s.	n.s.	n.s.	n.s.
	NTRC D3								n.s.	n.s.	n.s.
	LM10 D1									n.s.	n.s.
	LM10 D2										n.s.
	LM10 D3										

Table S7 (continued)

		Sham			Rotenone						
		Vehicle	NTRC D3	LM10 D3	Vehicle	NTRC D1	NTRC D2	NTRC D3	LM10 D1	LM10 D2	LM10 D3
Intestinal transit (Fig. 7A)											
Sham	Vehicle		n.s.	n.s.	< 0.0001	n.s.	< 0.001	n.s.	n.s.	n.s.	n.s.
	NTRC D3			n.s.	< 0.001	n.s.	< 0.01	n.s.	n.s.	n.s.	n.s.
	LM10 D3				< 0.001	n.s.	< 0.001	n.s.	n.s.	n.s.	n.s.
Rotenone	Vehicle					n.s.	n.s.	< 0.0001	n.s.	< 0.0001	< 0.0001
	NTRC D1						n.s.	n.s.	n.s.	n.s.	n.s.
	NTRC D2							< 0.0001	n.s.	< 0.0001	< 0.0001
	NTRC D3								n.s.	n.s.	n.s.
	LM10 D1									n.s.	n.s.
	LM10 D2										n.s.
	LM10 D3										
Colon length (Fig. 7B)											
Sham	Vehicle		n.s.	n.s.	< 0.0001	n.s.	n.s.	n.s.	n.s.	n.s.	n.s.
	NTRC D3			< 0.05	< 0.0001	< 0.01	< 0.01	n.s.	< 0.01	< 0.01	< 0.01
	LM10 D3				< 0.001	n.s.	n.s.	n.s.	n.s.	n.s.	n.s.
Rotenone	Vehicle					n.s.	n.s.	< 0.05	n.s.	< 0.05	< 0.05
	NTRC D1						n.s.	n.s.	n.s.	n.s.	n.s.
	NTRC D2							n.s.	n.s.	n.s.	n.s.
	NTRC D3								n.s.	n.s.	n.s.
	LM10 D1									n.s.	n.s.
	LM10 D2										n.s.
	LM10 D3										
GFAP expression (Fig. 7C)											
Sham	Vehicle		n.s.	n.s.	< 0.0001	< 0.0001	n.s.	n.s.	< 0.0001	< 0.0001	n.s.
	NTRC D3			n.s.	< 0.0001	< 0.0001	n.s.	n.s.	< 0.0001	< 0.0001	n.s.
	LM10 D3				< 0.0001	< 0.0001	n.s.	n.s.	< 0.0001	< 0.0001	n.s.
Rotenone	Vehicle					n.s.	< 0.0001	< 0.0001	n.s.	< 0.001	< 0.0001
	NTRC D1						n.s.	n.s.	n.s.	n.s.	n.s.
	NTRC D2							n.s.	< 0.01	n.s.	n.s.
	NTRC D3								< 0.01	n.s.	n.s.
	LM10 D1									< 0.01	< 0.001
	LM10 D2										n.s.
	LM10 D3										
α-Synuclein expression (Fig. 7D)											
Sham	Vehicle		n.s.	n.s.	< 0.05	n.s.	n.s.	n.s.	n.s.	n.s.	n.s.
	NTRC D3			n.s.	< 0.05	n.s.	n.s.	n.s.	n.s.	n.s.	n.s.
	LM10 D3				< 0.05	n.s.	n.s.	n.s.	n.s.	n.s.	n.s.
Rotenone	Vehicle					< 0.05	< 0.01	< 0.01	< 0.01	< 0.001	< 0.001
	NTRC D1						n.s.	n.s.	n.s.	n.s.	n.s.
	NTRC D2							n.s.	n.s.	n.s.	n.s.
	NTRC D3								n.s.	n.s.	n.s.
	LM10 D1									n.s.	n.s.
	LM10 D2										n.s.
	LM10 D3										
